# Clinical recommendations on diagnosis and treatment of immune checkpoint inhibitor‐induced renal immune‐related adverse events

**DOI:** 10.1111/1759-7714.13405

**Published:** 2020-03-30

**Authors:** Ke Zheng, Wei Qiu, Hanping Wang, Xiaoyan Si, Xiaotong Zhang, Li Zhang, Xuemei Li

**Affiliations:** ^1^ Department of Nephrology, Peking Union Medical College Hospital Chinese Academy of Medical Sciences Beijing China; ^2^ Department of Respirology, Peking Union Medical College Hospital Chinese Academy of Medical Sciences Beijing China; ^3^ Department of Internal Medicine, Peking Union Medical College Hospital Chinese Academy of Medical Sciences Beijing China

**Keywords:** Acute kidney injury, acute tubulointerstitial nephritis, immune checkpoint inhibitors, immune‐related adverse events

## Abstract

Immune checkpoint inhibitors (ICIs) are nowadays widely used in clinical oncology treatment, and significantly improve the prognosis of cancer patients. However, overactivation of T cells and related signaling pathways caused by ICIs can also induce immune‐related adverse effects (irAEs). Renal immune side‐effects are relatively rare, but some are serious and fatal. Acute kidney injury (AKI), diagnosed mainly by percentage increases in serum creatinine (sCr), is the most common clinical manifestation, while acute tubulointerstitial nephritis (ATIN) is the main cause of ICI‐related AKI. Urinalysis analysis and sediment evaluation, 24 hour urine protein and sCr are helpful in screening and monitoring renal irAEs. Multiple potential causes for AKI are involved during cancer therapy, and should be differentiated from the immune mechanisms of ICIs. Under these circumstances, a renal biopsy should be considered which is essential for clinical decision‐making. Steroids are an effective treatment option for renal irAEs. Most patients who experience ICI‐related ATIN achieve a partial or complete renal recovery with prompt diagnosis and treatment. Multidisciplinary collaborations of different specialists will improve the effectiveness and outcome in the management of ICI irAEs.

## Introduction

In recent years, studies on immune checkpoint inhibitors (ICIs) have greatly accelerated the development of oncology treatment. The principal mechanism of ICIs is the blockade of immune checkpoints, including cytotoxic T lymphocyte‐associated antigen‐4 (CTLA‐4) on the surface of T cells (eg, ipilimumab and tremelimumab) and programmed cell death protein 1 (PD‐1) receptor/programmed cell death ligand 1 (PD‐L1) pathway (eg, nivolumab and pembrolizumab), reactivating quiescent T cells inactive in the tumor microenvironment, thereby enabling them to resume their antitumor property and ability to mediate tumor cell death.^1^ However, overactivation of T cells may at the same time also induce immune‐related adverse events (irAE) to nontumor tissue.

Renal irAEs used to be considered relatively rare, but they can be serious, sometimes even causing death.^2^ In addition to the above mechanism of ICI irAEs, it is thought that ICIs can reactivate previously silenced drug‐specific T cells primed by nephritogenic drugs (proton pump inhibitors and nonsteroidal anti‐inflammatory drugs) associated with acute tubulointerstitial nephritis (ATIN), and consequently activate relative memory T cells against the drug.^3^ Another possible mechanism is that PDL‐1 is expressed in the kidney tubules, but not in the glomeruli, and therefore tubules are the mainly affected part of the kidney.^4^ Kidney function is prerequisite to successful cancer treatment. It is vital for clinicians to be familiar with the clinical and pathological manifestation of ICI‐related renal irAEs, as this will help to improve the diagnostic efficiency and optimize treatment strategy.

## Epidemiology

Acute kidney injury (AKI) is a clinical syndrome, defined as an abrupt decrease in kidney function which encompasses injury and impairment, and ICI‐related renal irAEs are usually determined to be as a result of ICI administration. However, other known causes of AKI should first be excluded. Based on a pooled analysis of 3695 patients treated with ICIs, reported in 2016 by Cortazar *et al*.^5^ the overall incidence of AKI was 2.2%, while the incidence of severe AKI was 0.6%, which included grade 3 AKI (an increase in SCr > 3‐fold above baseline, or an SCr >353.6 μmol/L (4.0 mg/dL) and grade 4 AKI (an increase in SCr > 6‐fold above baseline, or the need for renal replacement). Also, in their report, the incidence of renal irAEs was 2.0% with monotherapy of ipilimumab, and 1.9% with nivolumab. In a review by Manohar *et al*. in 2019 on programmed cell death protein 1 (PD‐1) inhibitor, 11 482 patients enrolled into the study demonstrated a similar pooled incidence of AKI with PD‐1 inhibitors, total AKI incidence was 2.2%, the pooled incidence of AKI with nivolumab treatment was 2.3% and with pembrolizumab was 2.0%.^6^

In a recently published study by Seethapathy *et al*. 1016 patients received ICI therapy at Massachusetts General Hospital, although the incidence of ICI‐related AKI was 2.95% which is similar to other reports, but surprisingly there was 17% patients who experienced AKI (defined as ≥1.5‐fold increase in creatinine from baseline), and 8.07% experienced sustained AKI (defined as an AKI event which lasted ≥3 days).^7^

A combination of ipilimumab and nivolumab has been reported to significantly increase the incidence of renal irAEs to 4.9%.^5^ Higher dosage or frequency has also been reported by Izzedine *et al*. to lead to a higher incidence of renal irAEs,^8^ while in the meta‐analysis of Manohar *et al*. by meta‐regression, there was no significant impact of dose of PD‐1 inhibitors (both nivolumab and pembrolizumab) on the rates of AKI, apart from a higher incidence of hypocalcemia in patients treated with high‐dose PD‐1 inhibitors.^6^

The onset time of renal irAEs is highly variable, ranging from weeks to months. Renal irAEs may occur after a single dose of ICI or several months after ICI treatment.^9^ The median symptom onset time has been reported to be three months.^5^ It has also been reported that most renal irAEs develop between 6–12 weeks after ipilimumab administration, while six to 12 months after nivolumab administration, and biopsy‐proven AIN has been reported to range from one to 12 months after pembrolizumab initiation.^10^

Unlike severe AKI, mild rises in creatinine are usually nonsymptomatic and sometimes difficult to notice without confirmation by laboratory test. At the same time, multiple reasons can cause fluctuations in kidney function in cancer patients who are receiving antitumor treatment. These make the diagnosis of renal irAEs more challenging and the precise incidence more difficult to determine. To date, based on limited data of kidney biopsies in patients received ICIs, it is also difficult to determine the accurate incidence of ICI‐related ATIN. However, we are of the opinion that with a better understanding of ICIs and a more comprehensive diagnosis and follow‐up system, a more accurate incidence of renal irAEs will be revealed in the near future.

## Renal pathological features

Acute tubulointerstitial nephritis (ATIN) is the most commonly reported pathological lesion in patients who have received ICI therapy.^5^ To date, the ability of many ICIs to cause acute tubulointerstitial damage has been widely reported.^10^, ^11^ Typical ICI‐related AIN appears as lymphocytic infiltration in the renal interstitium with or without granuloma, accompanied by varying degrees of plasma cells and/or eosinophil infiltration. Inflammatory cell infiltration can be diffuse or localized. Inflammatory cells can also infiltrate into the tubule, resulting in tubulitis and tubular basement membrane (TBM) rupture.^9^ Immunofluorescence (IF) studies are usually negative in ATIN.

There have been sporadic reports of other kinds of renal parenchymal damage, including lupus‐like immune complex glomerulonephritis in patients treated with ipilimumab,^12^ minimal change disease (MCD) in patients treated with pembrolizumab or ipilimumab,^13^, ^14^ and thrombotic microangiopathy in patients treated with pembrolizumab.

## Clinical features and management

In accordance with the prevalent pathological lesions in patients being treated with ICIs, tubular lesion‐related clinical manifestations are the main clinical findings in practice. In patients with ATIN, the clinical presentations may be nonspecific, while elevation of serum creatinine (sCr) is observed in almost all patients, accompanied with low‐grade proteinuria and sterile pyuria, and occasionally mild serum eosinophilia has also been reported to occur in some cases.^1^ Electrolyte abnormalities, including hyponatremia (which may relate to hypophysitis), hypokalemia, and hypocalcemia (mainly found in pembrolizumab), have also been seen in some cases. A case of hypocalcemia with undetectable PTH levels reported recently indicated that the change of serum calcium might be related to hypoparathyroidism in ICI therapy.^15^ Nephrotic syndrome and hypertension were not reported to be unusual, unless patients who developed MCD had nephrotic‐range proteinuria^13^, ^14^ and severe kidney function decline with secondary hypertension. In the very rare cases that developed immune‐complex glomerulonephritis, hematuria or active urine sediments were revealed. However, it should be noted, through a careful review of medication history, that proton pump inhibitors (PPIs) and nonsteroidal antiinflammatory drugs (NSAIDs) are common concurrent or prior medication in patients with renal irAEs.

Urine analysis, urine sediment, 24 hour urine protein and sCr are the most important and convenient screening/monitoring indicators. Based on the urine protein and sCr level, clinicians can make more rational clinical decisions (Table 1 and Table 2). Similar to the principle of management of general kidney diseases, for patients with proteinuria over 3.5 g/24 hours or recurrent proteinuria of 1–3.5 g/24 hours, kidney biopsy should be considered (Table 2). Kidney function should be monitored routinely at baseline and no less than three to six months after initiation of a PD‐1 inhibitor, and even sooner with patients who have received CTLA‐4 inhibitor. The causes of AKI are always complicated during the period of antitumor treatment. Before the exclusion of other causes of AKI, corticosteroids should not be initiated. The differential diagnosis of AKI should include prerenal causes (eg, dehydration) and ATN, post‐renal causes (eg, urinary tract obstruction) and renal parenchyma damage (eg, infection, nephrotoxin exposure and kidney disease with known reasons). A careful medical history should include volume status evaluation (fluid intake, urine output, diarrhea), infectious symptoms and concurrent medications (especially the administration of NSAIDs, PPIs and other nephrotoxic medications). Urine analysis and sediment, blood biochemistry test including kidney function indicators and electrolytes, and ultrasound examination of the urinary system should be performed. Urinary tract infection should first be ruled out if urinanalysis indicates pyuria or hematuria. If a patient has a history of mild hematuria, proteinuria and decreased kidney function at baseline, pre‐existing renal disease (eg, primary glomerulonephritis, hypertensive nephropathy and diabetic kidney disease) should be considered, a nephrology consultation is suggested and a more frequent check‐up on kidney function should be carried out. According to the NCCN2019 guidelines (Table 3), in the circumstance of AKI (sCr increase to 1.5‐fold of baseline or an increase of 0.3 mg/dL), sCr and urine protein should be evaluated at least every three to seven days, and clinicians alerted to potential complications related to kidney failure.

**Table 1:** Recommendations on management of ATIN with ICI therapy

Conditions	Work‐up	ICI management	Treatment
sCr 1–1.5 × baseline UPro≥2+ Leukocyturia (>5 WBC/HPF)	Rule out other causes Repeat tests 1 week later	Continue	Discontinue potential nephrotoxic drugs Correct prerenal AKI causes, etc.
sCr 1.5–3.0 × baseline UPro≥2+ Leukocyturia (>5 WBC/HPF)	Rule out other causes Consider kidney biopsy	Withhold ICIs	Discontinue potential nephrotoxic drugs When kidney biopsy confirms ATIN or empirical: Prednisolone 0.5–1.0 mg/kg/day or equivalent and continue until improvement to mild. Steroids taper over 1 month
sCr>3.0 × baseline UPro≥2+ Leukocyturia (>5 WBC/HPF)	Rule out other causes Perform kidney biopsy	Withhold ICIs	Discontinue potential nephrotoxic drugs When kidney biopsy confirms ATIN: Prednisolone 1.0–2.0 mg/kg/day or equivalent and continue until improvement to mild. Steroids taper over 1 months

ICIs, immune‐checkpoint inhibitors; sCr, serum creatinine; UPro, urinary protein.

**Table 2:** Recommendations on management of proteinuria with ICI therapy

Conditions	Work‐up	ICI management	Treatment
UPro < 1 g/24 hours	Rule out other causes Check sCr, urine analysis and sediment	Continue	Observe, if no active urine sediment
UPro 1–3.5 g/24 hours	Rule out other causes Check sCr, urine analysis and sediment Consider kidney biopsy	When kidney biopsy confirms: Withhold ICIs	Treat the diagnosed glomerular disease
UPro >3.5 g/24 hours	Kidney biopsy	When kidney biopsy confirms: Withhold ICIs	Treat the diagnosed glomerular disease

ICIs, immune‐checkpoint inhibitors; sCr, serum creatinine; UPro, urinary protein.

**Table 3:** Management of AKI (NCCN 2019 V2 management of immunotherapy‐related toxicities)

Conditions	Work‐up	Management
Mild (Grade 1)	sCr 1–1.5 × baseline or increase 0.3 mg/day(26.52 μmol/L)	Withhold ICIs Correct dehydration, withdraw nephrotoxic medication, Monitor sCr and Upro at least every 3–7 days
Moderate (Grade 2)	sCr 1.5–3 × baseline	Withhold ICIs Monitor sCr and Upro at least every 3–7 days Rule out other causes, correct dehydration, withdraw nephrotoxic medication Nephrology consultation Start prednisolone 0.5–1.0 mg/kg/day; For persistent G2 > 1 week, prednisolone 1.0–2.0 mg/kg/day
Severe (Grade 3)	sCr >3 × baseline or > 4 mg/dL (353.6 μmol/L)	Permanently discontinue ICIs Consider inpatient care Nephrology consultation and renal biopsy Start prednisolone 1.0–2.0 mg/kg/day
Life‐threatening (Grade 4)	sCr >6 × baseline or dialysis indicated	Initiate treatment with intravenous methylprednisolone; If >G2 after 1 week of steroids, consider other immunosuppressive therapy (MMF, CTX, AZA, infliximab)

AZA, azathioprine; CsA, cyclosporine; CTX, cyclophosphamide; MMF, mycophenolat; sCr, serum creatinine.

Although sCr is not the most sensitive measurement of kidney function, it is thought to be a cost‐effective screen test for AKI. Kidney biopsy is the golden standard in the diagnosis of kidney damage. It is essential to determine the cause and severity of kidney injury. A prompt kidney biopsy will contribute to a more rational adjustment in a treatment plan. Early detection of ICI‐related immune renal injury is vital so that steroid treatment can be initiated which is important to ensure a complete recovery of renal function. Even though some guidelines state that steroids can be initiated without kidney biopsy results in cases of suspected ICI‐related renal irAEs and before other causes of AKI can be eliminated, as nephrologists, we still strongly recommend a kidney biopsy to ensure that renal irAE is the major cause of AKI in patients as there are multiple reasons for AKI. A nephrology consultation should be considered in patients with toxicity higher than grade 1 and recurrent nephritis. Multidisciplinary teamwork and discussion are important in deciding whether to proceed with an invasive procedure, timing of ICI withdrawal, and ICI rechallenge.

## Treatment strategy

If there is any evidence of kidney damage during ICI therapy, the potential causes should be investigated immediately. Correcting the potential causes of AKI, including infection, dehydration and urinary tract obstruction is vital. All potential nephrotoxins should be withdrawn, particularly PPIs and NSAIDs, which could also induce ATIN and are presumed to be involved in the development of ICI renal irAEs.^16^ Aminoglycosides and contrast agents should also be avoided during this period.

Withholding treatment with ICIs should be considered if a patient develops AKI with sCr increase over 1.5‐fold of baseline, until the creatinine recovers to grade 1.

Corticosteroids should be initiated when a diagnosis of a renal irAEs is highly suspected in anyone with a grade 2 event or higher, and other causes of AKI have been excluded. To be noted, as nephrologists, we still strongly recommend a renal biopsy to confirm the renal irAE, which might also assist with an accurate evaluation of the patient's kidney's status for cancer therapy in the future.

Steroid treatment can be initiated with oral corticosteroids (prednisone or prednisolone) 0.5–1 mg/kg per day (up to 60 mg per day) which is similar to the treatment for allergic ATIN. For grade 3–4 disease, methylprednisolone intravenously might be considered for a short period. The duration of steroid treatment and the speed of taper is still not unified, but most studies support a treatment plan of prednisone maintained with 0.5–2 mg/kg for 1–2 months (depending on the initial sCr level and sCr improvement) followed by tapering with 5–10 mg/per week. Most patients respond well to steroids alone. For the few patients with a recurrence of AKI or those where it is difficult to wean them off steroids, other immunosuppressants (mycophenolate mofetil, cyclophosphamide and azathioprine) could be considered, but we still need more clinical data to set a suggestion on the most appropriate dosage and duration about these immunosuppressants. There have been reports on the use of infliximab in patients with renal irAEs for concomitant refractory colitis which indicate the treatment prospects for anti‐TNF‐α agents in renal irAEs.^9^

With grade 4 renal irAEs, renal replacement therapy should be administered to patients, with consideration given to dialysis. Dialysis treatment should make allowance for steroid therapy, and be adjusted according to the recovery rate of kidney function. As suggested by the general principle of management for ICI irAE, when grade 4 toxicities occur, or grade 2 toxicities last longer than six weeks, or there is difficulty in reducing the steroid dosage in a patient, ICIs should be permanently discontinued.^17^ In the case of biopsy‐proven ATIN with severe AKI, permanent discontinuation of ICIs should also be considered.

Rechallenge of ICI rechallenge should be applied with caution in patients whose creatinine recovers to normal or baseline rapidly (at least grade 1) on steroids.^9^ After starting ICI retreatment, blood tests should be repeated weekly for the first few months in order to detect and monitor any rise in creatinine levels.

## Prognosis

Steroids appear to be effective in most cases of renal irAEs. With an accurate judgement and prompt treatment, most patients with ICI‐related immuno‐ATIN obtain a complete or partial recovery of renal function.^5^ Evidence of granuloma formation on kidney biopsy may indicate a poor response to steroids and subsequent recovery of kidney function.

It has been reported that the resolution median time of renal‐selected events in nivolumab pivotal trials in squamous NSCLC was 5.9 weeks, ranging from 0.7 to 37.6 weeks,^8^ and the resolution time of grade 3–4 renal irAEs was 4.7 weeks (three to six weeks).^18^

In spite of limited available data, Harmankaya *et al*. report an antitumor response to ipilimumab with continued tumor regression more than two years following the last dose, even under the immunosuppressive effects of steroid therapy,^19^ and a retrospective study on ipilimumab also showed a similar overall survival in patients who received systemic immunosuppression.^20^ However, for patients with immune checkpoint inhibitor rechallenge, there could be recurrent AKI, and therefore more cautious monitoring of kidney function is reasonable.

## Conclusions

The introduction of ICIs into the treatment of cancer has significantly changed the concept of oncotherapy, and they are nowadays more widely used. In the meantime, immune‐related adverse events caused by overactivity of T cells in ICI treatment should receive more attention. The exact incidence of renal irAEs may be far beyond what is already known, especially for ICI‐related ATIN. Higher dosage and combination of ICIs may be relative to more renal irAEs. The challenges of AKI and ATIN in ICI therapy highlight the importance of kidney biopsy in renal irAEs, especially for patients with renal failure and/or nephrotic‐range proteinuria. Early detection of renal irAEs is vital, in order that steroid treatment can be administered to secure a complete recovery of renal function. The role of steroid‐sparing agents in immunosuppressive therapy of renal irAEs needs to be studied further in the future. With multidisciplinary collaboration, there will be a better understanding and better outcome for ICI‐related irAEs.

## Funding

This study has received funding by the Chinese Academy of Medical Science (CAMS) Initiative for Innovative Medicine (2017‐I2M‐2‐001, 2016‐I2M‐1‐002).

### Disclosure

The authors report there are no potential conflicts of interest.
